# Nutrients against Glucocorticoid-Induced Muscle Atrophy

**DOI:** 10.3390/foods11050687

**Published:** 2022-02-25

**Authors:** Min-Kyeong Lee, Hyeon Hak Jeong, Myeong-Jin Kim, Heeyeon Ryu, Jiwon Baek, Bonggi Lee

**Affiliations:** Department of Food Science and Nutrition, Pukyong National University, Nam-gu, Daeyeon-dong, Busan 48513, Korea; 3633234@hanmail.net (M.-K.L.); wjdgusgkr123@nate.com (H.H.J.); audwlsabc@naver.com (M.-J.K.); heeyeon3115@naver.com (H.R.); loog_ood@naver.com (J.B.)

**Keywords:** skeletal muscle, atrophy, steroid, glucocorticoid, nutrition

## Abstract

Glucocorticoid excess is a critical factor contributing to muscle atrophy. Both endogenous and exogenous glucocorticoids negatively affect the preservation of muscle mass and function. To date, the most effective intervention to prevent muscle atrophy is to apply a mechanical load in the form of resistance exercise. However, glucocorticoid-induced skeletal muscle atrophy easily causes fatigue in daily physical activities, such as climbing stairs and walking at a brisk pace, and reduces body movements to cause a decreased ability to perform physical activity. Therefore, providing adequate nutrients in these circumstances is a key factor in limiting muscle wasting and improving muscle mass recovery. The present review will provide an up-to-date review of the effects of various nutrients, including amino acids such as branched-chain amino acids (BCAAs) and β–hydroxy β–methylbutyrate (HMB), fatty acids such as omega-3, and vitamins and their derivates on the prevention and improvement of glucocorticoid-induced muscle atrophy.

## 1. Introduction

Skeletal muscle is the largest metabolic organ (40~50% of the body mass) and protein reservoir (50~75% of all body proteins) in the human body [1]. It is involved not only in controlling body movement but also in energy expenditure, respiration, and glucose, lipid, and amino acid homeostasis [1]. Skeletal muscle mass is an important determinant of muscular strength, muscular endurance, and the ability to perform physically. Muscle atrophy is the physiological result of aging, defined as the presence of both a decrease in skeletal muscle mass and muscle function (strength or performance), but it may also be caused by prolonged fasting, a sedentary lifestyle, diabetes, cachexia, sepsis, burns, and glucocorticoid excess [2,3]. Regardless of the cause, skeletal muscle atrophy contributes to a loss of independence and increases the risk of falls, ultimately resulting in a reduced quality of life [4,5,6]. In addition, muscle atrophy is one of the most important diseases to be solved to lead a healthy life in an aging society because it causes various socio-economic problems such as increased physical disability and mortality and increased medical and nursing care expenses [7].

Natural and synthetic glucocorticoid is one of the widely prescribed therapeutic compounds for treating and controlling inflammatory, lymphoproliferative, autoimmune, and neuromuscular diseases [8]. Synthetic glucocorticoids used in therapy include hydrocortisone, prednisone, and dexamethasone [9]. The efficacy criterion for synthetic glucocorticoids is expressed as a hydrocortisone equivalent potency, which is useful in determining the dosage of each glucocorticoid [10]. Currently, the generally recommended glucocorticoid dose for adults is 15–25 mg hydrocortisone per day in 2 to 3 divided doses [9,10]. However, higher doses than recommended are used in diseases such as asthma or brain tumors [11,12]. According to a previous study, when a manual strength test was performed on asthma patients taking 40 mg or more of prednisone per day, hip flexor weakness was observed in 64% of patients. However, in the group of patients taking less than 30 mg per day, only one patient developed muscle weakness [12]. These results indicate that the high-dose administration of glucocorticoids may cause side effects in the muscular system [3,12,13,14,15]. Although few long-term clinical trials are available to reveal the relationship between chronic synthetic glucocorticoid intake as a therapeutic purpose and muscle atrophy as a side effect, some clinical conditions may induce muscle atrophy by endogenously produced glucocorticoids [16,17,18,19,20]. Insulin deficiency-mediated acute diabetes has been reported to stimulate glucocorticoid-induced muscle atrophy. In support of this, adrenalectomized mice are resistant to muscle atrophy in a streptozotocin-mediated diabetic mouse model [17]. A metabolic acidosis because of disrupted renal acid excretion derived from chronic kidney disease is also closely related to glucocorticoid-induced muscle protein degradation [21]. In addition, tumor growth is closely related to systemic inflammation and an increase in blood glucocorticoids [20]. Thus, during abnormal conditions, such as infection, cancers, diabetes, and chronic kidney disease, multiple factors including inflammatory mediators stimulate the production of the hypothalamic corticotropin-releasing hormone followed by the release of an adrenocorticotrophic hormone by the anterior pituitary into blood [20]. Then, the adrenocorticotrophic hormone stimulates the release of cortisol by the adrenal gland. Cortisol induces the loss of skeletal muscle by the breakdown of contractile proteins and the mobilization of amino acids [20].

**Figure 1:**
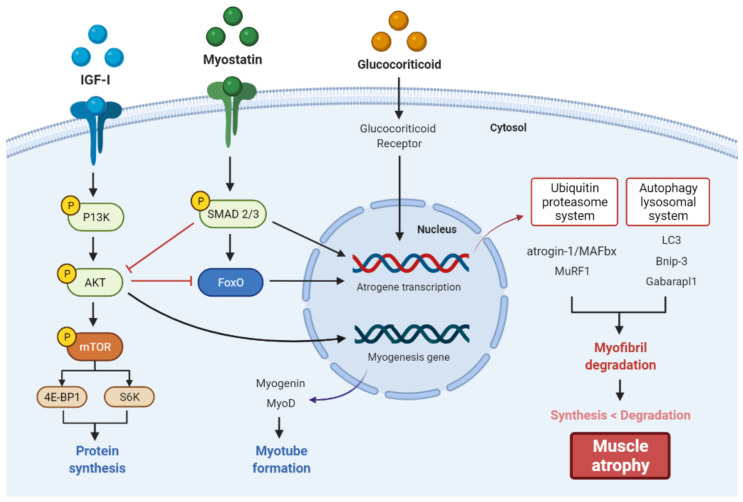
Molecular mechanisms of glucocorticoid-induced muscle atrophy. Glucocorticoid-induced skeletal muscle atrophy is associated with the altered expression of IGF-I and myostatin, two regulators that play important roles in skeletal muscle growth and development, resulting in decreased protein synthesis and increased proteolysis.

Glucocorticoid-induced muscle atrophy occurs when myofibrillar proteolysis exceeds protein synthesis and is characterized by a decrease in muscle fiber area or density [22]. In general, glucocorticoid-induced skeletal muscle atrophy has little or no effect on slow-twitch type I muscle fibers but is known to induce selective loss of fast-twitch type II muscle fibers [14]. This glucocorticoid-induced muscle proteolysis is mainly mediated by the activation of catabolic pathways, including the ubiquitin-proteasome and autophagy-lysosomal system [23]. Activation of these two proteolytic systems by glucocorticoid is mediated through the upregulated expression of muscle-specific E3 ubiquitin ligases, muscle atrophy F-box (MAFbx)/atrogin-1 and muscle RING finger-1 (MuRF1), and several autophagy genes, including microtubule-associated protein 1A/1B-light chain 3 (LC3), Bcl-2/adenovirus E1B 19-kDa-interacting protein 3 (Bnip-3), and GABA A receptor-associated protein-like 1 (Gabarapl1) [13,24,25]. Glucocorticoids can also cause muscle atrophy by locally affecting the expression of insulin-like growth factor-I (IGF-I), a positive regulator of muscle development, and Myostatin, a negative regulator of muscle development [26,27]. Glucocorticoids limit muscle protein synthesis by inhibiting the phosphorylation of the eukaryotic initiation factor 4E-binding protein 1 (4E-BP1) and ribosomal protein S6 kinase beta-1 (S6K1), which play essential roles in the protein synthesis machinery involved in the initiation of mRNA translation through the inhibition of the IGF-I/protein kinase B (Akt)/mammalian target of rapamycin (mTOR) pathway [28]. On the other hand, myostatin, a potent inhibitor of muscle growth, is released by glucocorticoids to stimulate phosphorylation of Smad2/3 and dephosphorylate Akt to increase ubiquitin-proteasome activity, leading to muscle atrophy [29]. These pathways are generally known to be regulated by specific transcription factors, such as forkhead box O (FoxO), which are phosphorylated and inactivated by Akt in the cytoplasm [29] (Figure 1).

Studies have shown that muscle atrophy may be caused by a sedentary lifestyle and an improper diet, such as low protein/high fat/low fiber, after approximately 30 years of age [30,31]. Fast-twitch skeletal muscle fiber atrophy by glucocorticoids easily causes fatigue from the activities of daily living, such as climbing stairs, walking at a brisk pace, and riding a bicycle, and this phenomenon becomes an obstacle to the patient’s rehabilitation treatment [32,33]. Additionally, the coronavirus disease 2019, which has persisted worldwide to date, has extended time at home by enforcing measures such as continued isolation and social distancing, resulting in decreased physical activity and altered dietary intakes that can accelerate body fat gain, deterioration of muscle mass and function, and sarcopenia [34]. Therefore, the importance of nutritional strategies to limit muscle wasting and improve muscle mass recovery is emerging in these circumstances [35,36,37,38]. The present review focuses on the current knowledge of nutrition-based therapies to counteract glucocorticoid-induced muscle atrophy based on cell and animal studies. However, very limited human studies were available about nutrients and glucocorticoid-induced muscle function. Thus, the general benefit of nutrients for muscle function in humans will be summarized in this review. Various articles have been obtained from Google Scholar and PubMed databases to provide a comprehensive analysis of the literature investigating the effects of nutrients on glucocorticoid-induced muscle atrophy. Literature searches were limited to English, and reference lists of all relevant studies and review articles were manually searched. Relevant keywords for the term glucocorticoid, muscle atrophy, and sarcopenia were analyzed in association with other terms such as “nutrition”, “amino acid”, “fatty acid”, “vitamin”, and “mineral”.

## 2. The Role of Nutrients

Nutrients in foods play a diverse role in maintaining energy homeostasis and tissue metabolism. They are also important for controlling myogenic differentiation and the functions of skeletal muscle. Various nutrients including amino acids, fatty acids, and vitamins and their derivates have been studied to investigate their effects on glucocorticoid-induced muscle atrophy.

### 2.1. Amino Acids

Various amino acids have been highlighted as a therapeutic factor for skeletal muscle diseases associated with muscle wasting (Table 1) [39,40]. A study investigated the utility of branched-chain amino acids (BCAA) in dexamethasone-related muscle atrophy using Sprague-Dawley rats. Intraperitoneal injection of dexamethasone reduced total protein levels and the mean cross-sectional area of soleus muscles and elevated the gene expression levels of *atrogin-1/MAFbx*, an essential factor to induce muscle atrophy, whereas the oral administration of BCAA reversed these characteristics [40]. In addition, dexamethasone-mediated conversion from LC3-I to LC3-II, a marker of autophagy, was inhibited through BCAA administration [40], indicating that BCAA reduced dexamethasone-induced protein disruption to suppress muscle atrophy.

Other studies investigated the effects of BCAA leucine supplementation or resistance exercise on dexamethasone-mediated muscle atrophy or insulin resistance using in vivo and in vitro models [39]. In vitro study using C2C12 myotubes showed that dexamethasone treatment notably inhibited protein synthesis and also decreased key signaling for protein metabolism as evidenced by a decrease in mTOR phosphorylation, ribosomal protein S6 protein kinase 1 (p70s6k1), and eukaryotic initiation factor 4E-BP1 [41]. However, leucine supplementation ameliorated protein synthesis and elevated mTOR phosphorylation possibly due to deactivating AMP-activated protein kinase (AMPK) signaling [41], a suppressor of protein synthesis in rat skeletal muscle [42], suggesting that leucine treatment may be beneficial for dexamethasone-induced disruption of protein synthesis in myotubes. An in vivo study using Sprague Dawley rats tested the effects of leucine and β–hydroxy β–methylbutyrate (HMB; a metabolite of leucine) in dexamethasone-induced muscle wasting [43]. The data showed that the oral administration of leucine or HMB significantly suppressed dexamethasone-mediated decreases in grip strength, muscle mass, and protein concentration in the soleus muscle [43]. It is assumed that the mechanisms underlying leucine or HMB-mediated protection of muscle wasting against dexamethasone involve the inactivation of FoxO1 transcription factor and subsequent down-regulation of MuRF1 expression [43].

However, controversial data were also reported in an animal study using dexamethasone-fed male Wistar rats that underwent resistance exercise and/or leucine supplementation through gavage [39]. Although resistance exercise ameliorated glucose tolerance in rats and elevated the phosphorylation of essential protein kinases (mTOR and p70S6k phospho/total ratio) and the transcription factors (FoxO3a phospho/total ratio) in skeletal muscle which are associated with resistance exercise-mediated muscle remodeling, leucine supplementation showed no therapeutic effects and worsened glucose homeostasis after dexamethasone administration [39]. Therefore, BCAA may have different therapeutic effects depending on the experimental conditions and BCAA dose, and further clinical trials are needed to conclude the consequence of BCAA supplementation for steroid-mediated muscle atrophy.

**Table 1:** The role of amino acids in glucocorticoid-induced muscle atrophy.

Name	Chemical Structures	Model	Effects	References
Branched-chain amino acids (BCAA leucine, valine, and isoleucine)	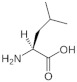 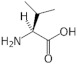 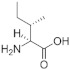	Sprague-Dawley rats	↑ total protein levels and mean cross-sectional area of soleus muscles ↓ *atrogin-1/MAFbx* ↓ conversion from LC3-I to LC3-II	[40]
Leucine	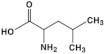	C2C12 myotubes	↓ AMPK signaling ↑ protein synthesis ↑ mTOR phosphorylation	[41]
		Sprague-Dawley rats	↑ grip strength, muscle mass, and protein concentration in soleus muscle (↓ FoxO1 transcriptional activity and ↓ MuRF1)	[43]
β–hydroxy β–methylbutyrate (HMB)	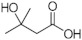	Sprague-Dawley rats	↑ grip strength, muscle mass, and protein concentration in soleus muscle (↓ FoxO1 transcriptional activity and ↓ MuRF1)	[43]

### 2.2. Clinical Relevance of Amino Acids in Muscle Atrophy

Few clinical trials have investigated the effects of amino acids on glucocorticoid-mediated muscle atrophy. Instead, many studies focused on the general effects of amino acid supplementation in healthy and elderly humans on muscle atrophy. Abnormal nutrition including underfeeding and obesity contributes to muscle atrophy. On the other hand, nutritional balance based on adequate exercise is a useful strategy to combat the muscle atrophy derived from various clinical muscle wasting conditions [44]. Various studies suggested that amino acid supplementation, especially essential amino acids, contributes to muscle protein synthesis in humans [45,46,47]. In particular, BCAA leucine supplementation improves muscle protein synthesis in the elderly by stimulating anabolic processes [48,49]. Additionally, when dietary protein intake was measured by a food-frequency questionnaire in the elderly (age 70–79), relatively high protein intake (1.1 g/kg/day) prevented the loss of lean mass compared to lower protein intake (0.7 g/kg/day) [50]. Further clinical trials are necessary to assess whether a protein intake higher than the recommended dietary allowance (0.8 g/kg/day) is beneficial for the elderly.

It appears that the total calorie intake may also be an important factor to elevate muscle mass in individuals with muscle-wasting conditions [44]. A study demonstrated that calorie-restricted individuals on bed rest showed excessive muscle loss [51], indicating the significance of energy availability to maintain muscle homeostasis. Moreover, an increase in protein intake without reducing other macronutrients contributes to increasing fat mass due to a positive energy balance, especially when physical activity was decreased [52]. These data indicate that the number of other nutrients and total calories needs to be considered when recommending extra protein intake to maintain or elevate muscle mass.

### 2.3. Fatty Acids

Polyunsaturated fatty acids (PUFAs) are used as health-promoting food supplements and are well-known to be particularly effective for the cardiovascular and central nervous systems [53]. The most consumed PUFAs are the omega-3 and omega-6 fatty acids that are widely studied and discussed in the scientific literature due to their beneficial functions in health [54]. Particularly, omega-3 fatty acids are commercialized worldwide as a food supplement with free access to the population in capsules. The omega-3 fatty acids such as docosahexaenoic acid (DHA), eicosapentaenoic acid (EPA), and α-linolenic acid (ALA) are incorporated into the cell membrane and regulate diverse cellular functions, such as protein signaling networks and gene expression (Table 2) [55].

A study demonstrated that DHA induces reactive oxygen species (ROS) production in C2C12 myotubes, leading to oxidative imbalance and the formation of protein aggregates [56]. In this study, DHA alone could not inhibit proteasome activity, but DHA-mediated protein aggregates could inhibit proteasome activity and delay protein degradation by blocking proteasome core particles [56]. Therefore, an in vitro study using C2C12 myotubes was conducted to confirm how these results affect muscle atrophy. In an in vitro model of dexamethasone (10 µM, 24 h)-induced muscle atrophy, DHA (25 µM) treatment increased the protein expression level of MyoD, a factor involved in the regulation of myotube formation. In addition, protein expression levels of atrogin-1 and LC3 factors related to myofibrillation were decreased. Consequently, DHA treatment was shown to prevent muscle wasting by inhibiting proteasome activity and delaying muscle protein degradation [56]. On the other hand, the effects of omega-6-fatty acids on glucocorticoid-induced muscle atrophy have not been extensively studied.

Although the beneficial effects of omega-3 fatty acids are documented in the skeletal muscle, its negative effects on dexamethasone-induced muscle wasting are also reported [54,57]. When the effects of the oral supplementation of omega-3 fatty acids on glucocorticoid-induced muscle atrophy were studied using Wistar rats [54], dexamethasone stimulated a notable decrease in body and muscle weight, atrophy in type 2B fibers, and reduced expression of p-Akt, p-GSK3β, and p-FoxO3a. Omega-3 fatty acid supplementation did not ameliorate the unfavorable effects of dexamethasone on skeletal muscle; actually, it induced atrophy in type 1, 2A, decreased the expression of myogenin, and elevated the expression of atrogin-1 [54]. Another study using Wistar rats investigated the effects of omega-3 on glucocorticoid-mediated muscle atrophy by considering the AMPK pathway beside the ubiquitin-proteasome and autophagy-lysosomal systems [57]. The supplementation of omega-3 fatty acid with dexamethasone treatment further elevated atrophy (fibers 1 and 2A), the mRNA expression of atrophy-associated genes such as *FoxO3a*, *p-SMAD2/3*, *atrogin-1/MAFbx*, and the protein markers of autophagy including LC3II, LC3II/LC3I, LAMP-1, and acid phosphatase [57]. Furthermore, omega-3 fatty acid administration alone reduced type 1 muscle fiber areas and the expression of PGC1-alpha and p-FoxO3a, indicating that omega-3 fatty acid worsens dexamethasone-mediated muscle atrophy partly by regulating AMPK signaling and an autophagic process [57]. Thus, the outcomes of omega 3 fatty acid supplementation in skeletal muscle may be different depending on the causal factors of muscle wasting such as biological aging, steroid hormones, etc. In addition, the unfavorable outcomes of omega-3 fatty acid supplementation have been reported in animal model studies in which muscle wasting occurred by dexamethasone treatment. Further clinical trials are necessary to conclude the consequence of omega-3 fatty acid supplementation on steroid-induced muscle wasting.

### 2.4. Clinical Relevance of Fatty Acids in Muscle Atrophy

When clinical studies were performed in younger [58] and older [59] human adults to investigate the effects of omega-3 fatty acid administration (8 weeks supplementation of 1.86 g/d of EPA and 1.50 g/d DHA) on the rates of mixed skeletal muscle protein synthesis in the fasted state and in response to a hyper-aminoacidemic-hyperinsulinemic infusion [58,59], DHA and EPA administration followed by subsequent incorporation into membrane phospholipids showed no effects on fasted rates of mixed muscle protein synthesis in response to the hyper-aminoacidemic-hyperinsulinemic infusion, whereas there was an enhancement of mixed muscle protein synthesis compared to before administration [58,59]. In addition, the enhancement of mixed muscle protein synthesis by omega-3 fatty acids was related to the elevation of mTORSer2448 and p70S6K1Thr389 phosphorylation in skeletal muscle [58,59]. Another human study in older adults exhibited that 1.86 g/d of EPA and 1.50 g/d DHA supplementation for 6 months significantly elevated muscle volume and strength in a free-living environment [60]. It appears that human studies suggest that the supplementation of omega-3 fatty acids elevates the rates of mixed muscle protein synthesis and skeletal muscle mass and size [58,59,60].

### 2.5. Vitamins

Vitamins play multi-functional roles in skeletal muscle metabolism. It has been reported that the vitamin-mediated antioxidative capacity and regulation of target genes associated with the functions of skeletal muscle play beneficial roles in steroid-induced muscle dysfunctions (Table 3) [61,62].

A recent study revealed that de novo expression of connexin43 and connexin45 hemichannels were involved in dexamethasone-induced muscle atrophy. When the outcomes of vitamin E supplementation on dexamethasone-mediated myotube atrophy were studied, dexamethasone increased the expression of connexin43 and connexin45, atrogin-1 immunoreactivity, oxidative stress, mitochondrial dysfunction, and atrophy in the skeletal muscle, whereas vitamin E supplementation reversed these characteristics [62]. Similarly, vitamin E suppressed connexin43/45 hemichannel activity in freshly isolated myofibers of dexamethasone-treated mice, suggesting that vitamin E is effective in relieving steroid-induced muscle atrophy at least partly by reducing connexin hemichannel activity [62]. The beneficial effects of vitamin E on muscle are also shown in broiler chickens [61]. Whilst dexamethasone inhibits the growth of chickens and elevates the formation of lipid peroxidation and the saturation level of fatty acids in the skeletal muscle, vitamin E (α-tocopherol)-fortified alleviated these phenomena, indicating that vitamin E is beneficial for the performance of broiler chickens at least partially by relieving the oxidative stress induced by dexamethasone [61].

Vitamin D is a fat-soluble vitamin and acts as a hormone via a nuclear receptor [63]. Previtamin D can be synthesized from 7-dehydrocholesterol by ultraviolet B irradiation in the skin and in turn be non-enzymatically converted to vitamin D through the transfer of double bonds. Vitamin D can be hydroxylated in the liver, producing 25-hydroxyvitamin D [25(OH)D]. Blood 25(OH)D concentration is used for measuring vitamin D insufficiency. The 25(OH)D is hydroxylated in the kidney, leading to the production of 1α,25-dihydroxy vitamin D [1,25(OH)2D] [63].

Several studies have demonstrated that vitamin D is beneficial in preventing sarcopenia [64] or inhibiting muscle atrophy [65], in part by suppression of FoxO1 transcriptional activity [66]. On the other hand, some studies also showed that there were little or no effects of vitamin D supplementation on muscle mass [67]. Furthermore, very limited research was performed to evaluate the effects of vitamin D on glucocorticoid-induced muscle atrophy. A study tested whether the abdominal injection of vitamin D3 for 4 weeks affects dexamethasone-mediated muscle atrophy using male Wistar rats [68]. Dexamethasone treatment decreased body weight compared to the control group throughout the experiment. Although vitamin D3 supplementation inhibited dexamethasone-induced body weight reduction, it significantly elevated soleus muscle mass despite no effect on extensor digitorum longus mass, indicating that vitamin D3 supplementation prevents dexamethasone-induced muscle loss, but only in red, not white, muscle [68]. Although further studies are necessary to reveal the mechanisms underlying vitamin D-mediated specific recovery of soleus muscle mass against dexamethasone, the authors speculate that vitamin D receptors in mitochondria are associated with this phenomenon. It has been well-known that vitamin D3 acts primarily through binding to intracellular vitamin D receptors, interacting with specific nucleotide sequences of over 60 target genes [68]. Furthermore, vitamin D receptors can be localized in the mitochondria and affect a decrease in mitochondrial respiration and serve in reprogramming cell metabolism toward the biosynthetic pathways [68,69,70], indicating the significance of mitochondria as a potential regulator linking the processes including cell development and the suppression of atrophy in skeletal muscles [68]. Thus, it is likely that vitamin D may be working more powerfully in soleus muscle consisting of mainly slow-oxidative fibers with a bigger pool of mitochondria compared to extensor digitorum longus consisting of primarily fast-glycolytic muscle fibers [68,71]. Another mechanism underlying beneficial effects of vitamin D on muscle atrophy include reduction in the expression of FoxO1 target atrophy genes including *atrogin-1* and *cathepsin L* in C2C12 myoblasts [66].

Retinoic acids (RA) are biologically active metabolites of vitamin A, and their biological effects are mediated through the nuclear receptors retinoic acid receptor (RAR) and retinoid x receptor (RXR). A study found that RA, the carboxyl form of vitamin A, reduces glucocorticoid sensitivity in C2C12 myotubes [72]. RA treatment in C2C12 myotubes significantly reduced the mRNA expression level of *11β-HSD1* in a time-dependent manner (2, 4, 10, and 24 h) and also inhibited GR transactivation in dexamethasone-treated C2C12 myotubes. These effects of RA may induce suppression of *11β-HSD1* mRNA expression and activity, thereby inhibiting GR transcriptional activation and reducing glucocorticoid sensitivity in skeletal muscle cells.

The roles of vitamin C in glucocorticoid-mediated muscle atrophy have not been extensively tested. Nevertheless, the general beneficial effects of vitamin C on muscle atrophy have been reported in an in vivo study using vitamin C-deficient senescence marker protein-30 (SMP30)-knockout (KO) mice [73]. Vitamin C-deficient mice had a markedly reduced muscle mass in the gastrocnemius, soleus, tibialis anterior, plantaris, and extensor digitorum longus muscles compared to mice supplemented with vitamin C at 12 and 18 weeks. Consistently, the cross-sectional area of the soleus muscle and physical performance measured by grip strength, treadmill, and home cage activity was significantly reduced in vitamin C-deficient SMP30 KO mice compared to SMP30 KO mice supplemented with vitamin C [73], indicating that vitamin C is pivotal to maintain skeletal muscle mass and physical activity performance. In the molecular signaling, the mRNA expression levels of ubiquitin ligases, such as *atrogin-1/MAFbx* and *MuRF1*, were elevated in SMP30 KO mice. On the other hand, all the effects reduced by vitamin C deficiency were restored by vitamin C supplementation in SMP30 KO mice, suggesting that vitamin C deficiency is closely related to muscle wasting and can be reversed by sufficient vitamin C supplementation [73].

**Table 3:** The role of vitamins in glucocorticoid-induced muscle atrophy.

Name	Chemical Structures	Model	Effects	References
Vitamin E	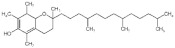	HeLa cells stably transfected with mouse Cx43 (HeLa-Cx43), or Cx45 (HeLa-Cx45)	↓ connexin43 and connexin45 ↓ atrogin-1 immunoreactivity ↓ oxidative stress ↓ mitochondrial dysfunction ↓ atrophy in the skeletal muscle	[62]
		Adult male mice (skeletal myofibers-deficient for Cx43 and Cx45 expression)	↓ connexin43/45 hemichannel activity	[62]
		Broiler Chicken	↑ growth of chickens ↓ lipid peroxidation ↓ saturation level of fatty acids in the skeletal muscle	[61]
Vitamin D	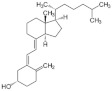	Wister rats	↓ FoxO1 transcriptional activity ↑ soleus muscle mass	[68]
		C2C12 myoblasts	↓ FoxO1 target atrophy genes (*atrogin-1*, *cathepsin L*)	[66]
Retinoic acid	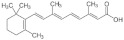	C2C12 myotubes	↓ *11beta-HSD1* ↓ GR transactivation	[72]
Vitamin C	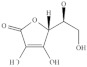	SMP30-KO mice	↑ muscle mass (gastrocnemius, soleus, tibialis anterior, plantaris, and extensor digitorum longus muscles) ↑ cross-sectional area of the soleus muscle ↑ physical performance (grip strength, treadmill, and home cage activity) ↓ ubiquitin ligases (*atrogin-1/MAFbx*, *MuRF1*)	[73]

### 2.6. Clinical Relevance of Vitamins in Muscle Atrophy

Although few studies were found about the effects of vitamin D on glucocorticoid-induced muscle atrophy, vitamin D supplementation considering 25(OH)D levels may be helpful to maintain muscle health in general. Based on the vitamin D recommendation (the Central European Recommendation) for healthy individuals and healthy individuals with a high risk of vitamin D deficiency, it is suggested to take vitamin D to achieve 25(OH)D levels in a range of 30–50 ng/mL [74,75]. There is a positive correlation between serum 25(OH)D levels and muscle function [76]. Additionally, vitamin D supplementation considering 25(OH)D levels has been reported to elevate muscle strength. A systemic review of 29 studies showed that vitamin D supplementation elevated muscle strength more in individuals with serum 25(OH)D levels at <30 ng/mL compared to those with >30 ng/mL, indicating that vitamin D supplementation may be more effective in individuals with low serum 25(OH)D levels [63,67].

Other clinical trials in older adults have found a positive correlation between vitamin status and muscle mass. One study investigated the effects of 6 months of cholecalciferol supplementation on muscle mass and strength in older adults after randomly assigning 128 vitamin D-deficient (25(OH)D = 12.92 ± 4.3 ng/mL) older adults (62 men and 66 women) to either a cholecalciferol-treated group (n = 64) or a placebo tablet-treated group (n = 64) [77]. One hundred fifteen participants completed the study, and the group taking 10,000 IU of cholecalciferol 3 times a week for 6 months significantly improved limb skeletal muscle mass, but not grip strength, compared to the placebo group [77]. The same study found that vitamin D-induced increases in appendicular skeletal muscle mass were higher in normal-weight subjects than in obese subjects [77]. In a study in postmenopausal women aged 50 to 65 years with vitamin D deficiency and a history of falls, daily administration of 1000 IU cholecalciferol for 9 months significantly increased lower extremity muscle strength compared to the placebo group [78]. In a trial of early postmenopausal women with vitamin D deficiency, 4000 IU ergocalciferol administered once a week for 12 weeks did not show notable differences in strength, muscle mass, or muscle CSA compared to the placebo group [79]. However, the group supplemented with ergocalciferol for 12 weeks had notable increases in muscle strength and muscle CSA compared to the baseline [79].

Overall, when thinking about vitamin D supplementation for muscle health, multiple factors should be considered to achieve better beneficial effects. Because vitamin D can be synthesized by ultraviolet B irradiation and further converted to 25(OH)D and 1α,25-dihydroxy vitamin D in the liver and kidney, respectively, the degree of UV exposure and the presence of renal or hepatic dysfunction should be considered to set up the proper dose of vitamin D. Moreover, vitamin D supplementation considering serum 25(OH)D levels has to be taken into account to maximize the beneficial effects of vitamin D for muscle function.

Oxidative stress is known as one of the major factors potentially implicated in the pathogenesis of sarcopenia [80]. ROS produced by oxidative stress are directly involved in muscle atrophy and the loss of skeletal muscle function and induce the expression of inflammatory cytokines such as TNF-α, IL-1, and IL-6 [80]. Therefore, well-known antioxidants, namely vitamins A, C, and E, can be an effective countermeasure to inhibit the development of sarcopenia by suppressing the production of ROS. A recent study evaluated the effect of vitamin E on muscle damage and inflammation induced by exercise performed under hypoxic conditions [81]. Nine healthy male volunteers were randomly assigned to perform three sessions of 60 min of exercise for 1 week in (i) normoxia, (ii) hypoxia, and (ii) hypoxia after acute administration of vitamin E (250 mg) 1 h before exercise [81]. The exercise in hypoxic conditions reduced hemoglobin oxygen saturation and increased blood concentrations of creatine kinase, lactate dehydrogenase, TNF-α, IL-1Rα, IL-6, and IL-10, but supplementation with vitamin E reversed these properties [81]. These results suggest that vitamin E supplementation may prevent muscle damage by attenuating oxidative stress and inflammation after exercise under hypoxia. Another study investigated the effects of 6 months of antioxidant vitamins (600 mg/day of vitamin E and 1000 mg/day of vitamin C) supplementation combined with resistance exercise on fat free-mass (FFM) and muscle mass index (MMI) in the elderly after randomly assigning 61 healthy older adults to a placebo-treated group, placebo-treated and resistance training group, antioxidant vitamins-treated group, and antioxidant vitamins-treated and resistance training group [82]. The researchers confirmed that the total FFM and MMI were markedly increased compared to the baseline in the group combining antioxidant vitamins with resistance training compared to the resistance training alone group [82]. Another study has demonstrated that intake of vitamin E at 600 mg/day and vitamin C at 1000 mg/day favorably altered the oxidation profile of athletes performing habitual training activities [83]. Taken together, these data suggest that vitamins C and E may enhance the effects of exercise on muscle mass gain through improved oxidation profiles. However, despite the fact that vitamins probably affect muscle atrophy, further research is needed to establish the extent to which vitamins can affect muscle.

### 2.7. Minerals

Minerals are metals and inorganic compounds, mostly supplied from the soil through plants to animals and humans [84]. They form most of the human skeleton such as bones and teeth, act as cofactors or activators of enzymes, and enable various essential activities of the body, such as the maintenance and function of cell membranes and secretion of hormones [84].

The roles of various minerals in glucocorticoid-mediated muscle atrophy have not yet been extensively tested. Nevertheless, it has recently been confirmed that sulforaphane, a sulfur compound, can inhibit dexamethasone-induced muscle atrophy through the Akt/FoxO pathway [85] (Table 4). An in vitro study using C2C12 myotubes indicated that dexamethasone treatment markedly activated proteolysis and up-regulated the mRNA expression of muscle-specific ubiquitin E3 ligases markers, such as *atrogin-1/MAFbx* and *myostatin* [85]. In addition, dexamethasone treatment inhibited protein synthesis, myotube diameter, and mRNA expression of *MyoD*, a factor regulating myogenesis, whereas supplementation with sulforaphane reversed these characteristics [85]. Furthermore, an anti-atrophic process that stimulates protein synthesis and reduces proteolysis in dexamethasone-induced C2C12 myotubes involves suppression of the transcriptional activity of FoxO1 by activation of Akt phosphorylation [85]. These results suggest that sulforaphane exhibits anti-muscle atrophy efficacy by promoting protein synthesis and inhibiting proteolysis through the Akt/FoxO1 pathway. However, further studies are needed to clarify the possible molecular mechanisms involved in sulforaphane-mediated anti-muscle atrophy action. Additionally, more research on the induction of muscle atrophy improvement by sulforaphane is needed using in vivo models of muscle atrophy.

### 2.8. Clinical Relevance of Minerals in Muscle Atrophy

To date, no clinical trials have been conducted to confirm the effect of minerals on glucocorticoid-induced muscle atrophy. An intervention study in some older adults found that selenium and magnesium supplementation positively affected muscle mass [86,87]. A study investigated the effects of 12 weeks of oral magnesium supplementation on physical performance in healthy older women after randomly assigning 124 healthy older women (71.5 ± 5.2 years) to either a magnesium-treated group (300 mg magnesium/day; n = 53) or a control group (no treatment; n = 71) [86]. The treatment group supplemented with magnesium for 12 weeks was markedly better than the control group in Short Physical Performance Battery (SPPB) scores, chair stand times, and four-minute walking speeds. Another study investigated the effect of magnesium supplementation of 250 mg for 8 weeks on muscle mass and strength in middle-aged, overweight women after randomly assigning 64 healthy middle-aged, overweight women to either a magnesium treated group or a placebo tablet treated group [88]. The group administered with 250 mg of magnesium daily for 8 weeks showed a notable decrease in fat mass with a notable increase in lean body mass compared to the baseline but did not show a significant change compared to the placebo tablet-treated group [88]. Grip strength and Time Get Up and Go Test (TGUG) were also improved in the magnesium-treated group compared to the baseline, but there were no notable changes compared to the placebo tablet-treated group, indicating that magnesium administration for 8 weeks did not markedly improve muscle mass and strength compared to placebo administration [88]. These findings suggest that magnesium supplementation may be beneficial for preventing or delaying aging-related decline in bodily functions.

Clinical trials of the elderly living in communities have shown that selenium supplementation may help improve health-related quality of life [87]. In a double-blind, randomized placebo-controlled trial, a combination of 200 µg/day organic selenium yeast and 200 mg/day CoQ10 or placebo was administered to 443 elderly subjects for four years [87]. A total of 206 older adults were evaluated after 4 years, and the selenium and CoQ10 combination group had fewer hospital days and significantly fewer decreases in physical role performance, vitality, and physical component scores compared to the placebo group [87].

Some studies have also shown associations between other minerals and muscle atrophy. Cross-sectional analysis from the UK Biobank found that higher potassium and calcium intakes were associated with a lower incidence of muscle atrophy [89]. Another case-control study that compared older adults with and without sarcopenia in a 1:1 ratio found that the older group with sarcopenia consumed markedly less phosphorus [90]. As noted earlier, although some studies have shown a significant association between muscle atrophy and dietary calcium and phosphorus intake, this is not the case for all. In a cross-sectional study of older adults, there was no significant difference in daily calcium intake between sarcopenic and non-sarcopenic older adults [91]. Another cross-sectional analysis of subjects 65 years of age and older found a significant inverse correlation between phosphate and muscle strength [92]. Therefore, the effect of the intake of minerals such as calcium and phosphorus on muscle mass preservation is unclear overall, and additional studies are required in the future to consolidate their link.

## 3. Conclusions

**Figure 2:**
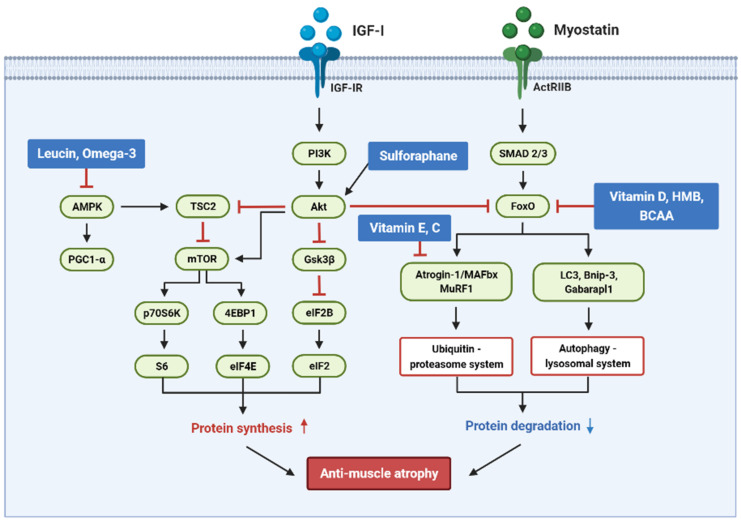
Anti-muscle atrophy signaling pathway regulated by multiple nutrients in a glucocorticoid-induced muscle atrophy model. The figure shows the major pathways that help maintain muscle protein turnover rate by inducing protein synthesis and inhibiting protein degradation.

Nutrients in food maintain energy homeostasis and tissue metabolism and play a critical role in regulating muscle differentiation and skeletal muscle function. Accumulated evidence supports that nutrients may help improve glucocorticoid-induced muscle atrophy through various mechanisms regulating the balance between protein synthesis and protein degradation, i.e., protein turnover. Figure 2 presents an overview of the targets of the signaling pathways of various nutrients that control glucocorticoid-induced muscular atrophy.

Various nutrients such as proteins, fatty acids, vitamins, and minerals are involved in the muscle protein metabolism by regulating the activity of IGF-I and myostatin, which are growth factors that play an essential role in controlling skeletal muscle mass. The studies covered in this review suggest that proteins, fatty acids, vitamins, and minerals may have the potential to modulate protein synthesis and degradation, mitochondrial metabolism, and energy production, which has the potential to affect muscle mass, strength, performance, and function. These results show the potential for nutrients to ameliorate glucocorticoid-induced muscle atrophy, but more extensive studies are still needed to support these results. Most studies have used cellular and animal models to evaluate the efficacy of nutrients in glucocorticoid-induced muscle atrophy. To the best of our knowledge, studies using humans as the main model do not exist. Therefore, there is a need to elucidate the underlying mechanisms of nutrient action on human muscle tissue and clarify how these changes translate into clinical effects. Additionally, clinical trials are required to define optimal dose-effect conditions for nutrients to improve skeletal muscle function.

## Figures and Tables

**Table 2:** The role of fatty acids in glucocorticoid-induced muscle atrophy.

Name	Model	Beneficial or Negative Effects	Effects	References
DHA	Dexamethasone-treated C2C12 myotubes	Beneficial effects	↑ protein expression level of MyoD ↓ protein expression levels of atrogin-1 and LC3 ↓ proteasome activity ↓ muscle protein degradation	[56]
Omega-3	Dexamethasone-treated Wistar rats	Negative effects	↑ type 1, 2A atrophy ↓ myogenin ↑atrogin-1/MAFbx	[54]
	Dexamethasone-treated Wistar rats	Negative effects	↑ atrophy associated genes (*FoxO3a, p-SMAD2/3, atrogin-1/MAFbx*) ↑ protein markers of autophagy (LC3II, LC3II/LC3I, LAMP-1, acid phosphatase) ↓ type 1 muscle fiber area ↓ PCG-1α, p-FoxO3a	[57]

**Table 4:** The role of minerals in glucocorticoid-induced muscle atrophy.

Name	Chemical Structures	Model	Effects	References
Sulforaphane	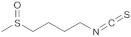	C2C12 myotubes	↑ protein synthesis ↓ protein degradation ↑ Akt phosphorylation activity ↓ FoxO1 transcriptional activity ↓ ubiquitin E3 ligases (*atrogin-1/MAFbx*, *MuRF1*) ↑ *MyoD*	[85]

## Data Availability

The datasets generated for this study are available on request to the corresponding author.

## References

[1] Baskin K.K., Winders B.R., Olson E.N. (2015). Muscle as a "mediator" of systemic metabolism. Cell Metab..

[2] Fanzani A., Conraads V.M., Penna F., Martinet W. (2012). Molecular and cellular mechanisms of skeletal muscle atrophy: An update. J. Cachexia Sarcopenia Muscle.

[3] Schakman O., Kalista S., Barbe C., Loumaye A., Thissen J.P. (2013). Glucocorticoid-induced skeletal muscle atrophy. Int. J. Biochem. Cell Biol..

[4] Bodine S.C. (2013). Disuse-induced muscle wasting. Int. J. Biochem. Cell Biol..

[5] Fry C.S., Rasmussen B.B. (2011). Skeletal muscle protein balance and metabolism in the elderly. Curr. Aging Sci..

[6] Larsson L. (2007). Experimental animal models of muscle wasting in intensive care unit patients. Crit. Care Med..

[7] Yeon M., Choi H., Jun H.S. (2020). Preventive Effects of Schisandrin A, A Bioactive Component of Schisandra chinensis, on Dexamethasone-Induced Muscle Atrophy. Nutrients.

[8] Nicolaides N.C., Charmandari E., Chrousos G.P., Kino T. (2014). Recent advances in the molecular mechanisms determining tissue sensitivity to glucocorticoids: Novel mutations, circadian rhythm and ligand-induced repression of the human glucocorticoid receptor. BMC Endocr. Disord..

[9] Trikudanathan S., McMahon G.T. (2008). Optimum management of glucocorticoid-treated patients. Nat. Clin. Pr. Endocrinol. Metab..

[10] Williams D.M. (2018). Clinical Pharmacology of Corticosteroids. Respir. Care.

[11] Dietrich J., Rao K., Pastorino S., Kesari S. (2011). Corticosteroids in brain cancer patients: Benefits and pitfalls. Expert Rev. Clin. Pharm..

[12] Bowyer S.L., LaMothe M.P., Hollister J.R. (1985). Steroid myopathy: Incidence and detection in a population with asthma. J. Allergy Clin. Immunol..

[13] Auclair D., Garrel D.R., Chaouki Zerouala A., Ferland L.H. (1997). Activation of the ubiquitin pathway in rat skeletal muscle by catabolic doses of glucocorticoids. Am. J. Physiol..

[14] Dekhuijzen P.N., Gayan-Ramirez G., Bisschop A., De Bock V., Dom R., Decramer M. (1995). Corticosteroid treatment and nutritional deprivation cause a different pattern of atrophy in rat diaphragm. J. Appl Physiol..

[15] Bodine S.C., Furlow J.D. (2015). Glucocorticoids and Skeletal Muscle. Adv. Exp. Med. Biol..

[16] Fitts R.H., Romatowski J.G., Peters J.R., Paddon-Jones D., Wolfe R.R., Ferrando A.A. (2007). The deleterious effects of bed rest on human skeletal muscle fibers are exacerbated by hypercortisolemia and ameliorated by dietary supplementation. Am. J. Physiol. Cell Physiol..

[17] Hu Z., Wang H., Lee I.H., Du J., Mitch W.E. (2009). Endogenous glucocorticoids and impaired insulin signaling are both required to stimulate muscle wasting under pathophysiological conditions in mice. J. Clin. Investig..

[18] Merritt E.K., Cross J.M., Bamman M.M. (2012). Inflammatory and protein metabolism signaling responses in human skeletal muscle after burn injury. J. Burn Care Res..

[19] Callahan L.A., Supinski G.S. (2009). Sepsis-induced myopathy. Crit. Care Med..

[20] Braun T.P., Marks D.L. (2015). The regulation of muscle mass by endogenous glucocorticoids. Front. Physiol..

[21] May R.C., Kelly R.A., Mitch W.E. (1986). Metabolic acidosis stimulates protein degradation in rat muscle by a glucocorticoid-dependent mechanism. J. Clin. Investig..

[22] Lofberg E., Gutierrez A., Wernerman J., Anderstam B., Mitch W.E., Price S.R., Bergstrom J., Alvestrand A. (2002). Effects of high doses of glucocorticoids on free amino acids, ribosomes and protein turnover in human muscle. Eur. J. Clin. Investig..

[23] Sandri M. (2013). Protein breakdown in muscle wasting: Role of autophagy-lysosome and ubiquitin-proteasome. Int. J. Biochem. Cell Biol..

[24] Foletta V.C., White L.J., Larsen A.E., Leger B., Russell A.P. (2011). The role and regulation of MAFbx/atrogin-1 and MuRF1 in skeletal muscle atrophy. Pflug. Arch..

[25] Penna F., Costamagna D., Pin F., Camperi A., Fanzani A., Chiarpotto E.M., Cavallini G., Bonelli G., Baccino F.M., Costelli P. (2013). Autophagic degradation contributes to muscle wasting in cancer cachexia. Am. J. Pathol.

[26] Gilson H., Schakman O., Combaret L., Lause P., Grobet L., Attaix D., Ketelslegers J.M., Thissen J.P. (2007). Myostatin gene deletion prevents glucocorticoid-induced muscle atrophy. Endocrinology.

[27] Yoshida T., Delafontaine P. (2020). Mechanisms of IGF-1-Mediated Regulation of Skeletal Muscle Hypertrophy and Atrophy. Cells.

[28] Liu Z.Q., Li G., Kimball S., Jahn L., Barrett E. (2004). Glucocorticoids modulate amino acid-induced translation initiation in human skeletal muscle. Am. J. Physiol. Endocrinol. Metab..

[29] Pereira R., De Carvalho J. (2011). Glucocorticoid-induced myopathy. Jt. Bone Spine Rev. Du Rhum..

[30] Vandewoude M., Alish C., Sauer A., Hegazi R. (2012). Malnutrition-Sarcopenia Syndrome: Is This the Future of Nutrition Screening and Assessment for Older Adults?. J. Aging Res..

[31] Liguori I., Russo G., Aran L., Bulli G., Curcio F., Della-Morte D., Gargiulo G., Testa G., Cacciatore F., Bonaduce D. (2018). Sarcopenia: Assessment of disease burden and strategies to improve outcomes. Clin. Interv. Aging.

[32] Lucia A., Nogales-Gadea G., Pérez M., Martin M., Andreu A., Arenas J. (2008). McArdle disease: What do neurologists need to know?. Nat. Clin. Pract. Neurol..

[33] Nogales-Gadea G., Consuegra-Garcia I., Rubio J.C., Arenas J., Cuadros M., Camara Y., Torres-Torronteras J., Fiuza-Luces C., Lucia A., Martin M.A. (2012). A transcriptomic approach to search for novel phenotypic regulators in McArdle disease. PLoS ONE.

[34] Woods J., Hutchinson N., Powers S., Roberts W., Gomez-Cabrera M., Radak Z., Berkes I., Boros A., Boldogh I., Leeuwenburgh C. (2020). The COVID-19 pandemic and physical activity. Sports Med. Health Sci..

[35] Magne H., Savary-Auzeloux I., Remond D., Dardevet D. (2013). Nutritional strategies to counteract muscle atrophy caused by disuse and to improve recovery. Nutr. Res. Rev..

[36] Wall B.T., van Loon L.J. (2013). Nutritional strategies to attenuate muscle disuse atrophy. Nutr. Rev..

[37] Marshall R., Smeuninx B., Morgan P., Breen L. (2020). Nutritional Strategies to Offset Disuse-Induced Skeletal Muscle Atrophy and Anabolic Resistance in Older Adults: From Whole-Foods to Isolated Ingredients. Nutrients.

[38] Shimizu M., Sakuma K., Julianna C. (2020). Nutritional Approaches for Attenuating Muscle Atrophy. Background and Management of Muscular Atrophy.

[39] Nicastro H., Zanchi N.E., da Luz C.R., de Moraes W.M.A.M., Ramona P., de Siqueira Filho M.A., Chaves D.F.S., Medeiros A., Brum P.C., Dardevet D. (2012). Effects of leucine supplementation and resistance exercise on dexamethasone-induced muscle atrophy and insulin resistance in rats. Nutrition.

[40] Yamamoto D., Maki T., Herningtyas E.H., Ikeshita N., Shibahara H., Sugiyama Y., Nakanishi S., Iida K., Iguchi G., Takahashi Y. (2010). Branched-chain amino acids protect against dexamethasone-induced soleus muscle atrophy in rats. Muscle Nerve.

[41] Wang X.J., Yang X., Wang R.X., Jiao H.C., Zhao J.P., Song Z.G., Lin H. (2016). Leucine alleviates dexamethasone-induced suppression of muscle protein synthesis via synergy involvement of mTOR and AMPK pathways. Biosci. Rep..

[42] Bolster D.R., Crozier S.J., Kimball S.R., Jefferson L.S. (2002). AMP-activated Protein Kinase Suppresses Protein Synthesis in Rat Skeletal Muscle through Down-regulated Mammalian Target of Rapamycin (mTOR) Signaling*. J. Biol. Chem..

[43] Noh K.K., Chung K.W., Choi Y.J., Park M.H., Jang E.J., Park C.H., Yoon C., Kim N.D., Kim M.K., Chung H.Y. (2014). β-Hydroxy β-methylbutyrate improves dexamethasone-induced muscle atrophy by modulating the muscle degradation pathway in SD rat. PLoS ONE.

[44] Owens D.J., Xiao J. (2018). Nutritional Support to Counteract Muscle Atrophy. Muscle Atrophy.

[45] Bennet W.M., Connacher A.A., Scrimgeour C.M., Smith K., Rennie M.J. (1989). Increase in anterior tibialis muscle protein synthesis in healthy man during mixed amino acid infusion: Studies of incorporation of [1-13C]leucine. Clin. Sci..

[46] Volpi E., Ferrando A.A., Yeckel C.W., Tipton K.D., Wolfe R.R. (1998). Exogenous amino acids stimulate net muscle protein synthesis in the elderly. J. Clin. Investig..

[47] Børsheim E., Tipton K.D., Wolf S.E., Wolfe R.R. (2002). Essential amino acids and muscle protein recovery from resistance exercise. Am. J. Physiol. Endocrinol. Metab..

[48] Rieu I., Balage M., Sornet C., Giraudet C., Pujos E., Grizard J., Mosoni L., Dardevet D. (2006). Leucine supplementation improves muscle protein synthesis in elderly men independently of hyperaminoacidaemia. J. Physiol..

[49] Katsanos C.S., Kobayashi H., Sheffield-Moore M., Aarsland A., Wolfe R.R. (2006). A high proportion of leucine is required for optimal stimulation of the rate of muscle protein synthesis by essential amino acids in the elderly. Am. J. Physiol. Endocrinol. Metab..

[50] Houston D.K., Nicklas B.J., Ding J., Harris T.B., Tylavsky F.A., Newman A.B., Lee J.S., Sahyoun N.R., Visser M., Kritchevsky S.B. (2008). Dietary protein intake is associated with lean mass change in older, community-dwelling adults: The Health, Aging, and Body Composition (Health ABC) Study. Am. J. Clin. Nutr..

[51] Bergouignan A., Momken I., Schoeller D.A., Normand S., Zahariev A., Lescure B., Simon C., Blanc S. (2010). Regulation of energy balance during long-term physical inactivity induced by bed rest with and without exercise training. J. Clin. Endocrinol. Metab..

[52] Forbes G.B., Brown M.R., Welle S.L., Lipinski B.A. (1986). Deliberate overfeeding in women and men: Energy cost and composition of the weight gain. Br. J. Nutr..

[53] Delgado-Lista J., Perez-Martinez P., Lopez-Miranda J., Perez-Jimenez F. (2012). Long chain omega-3 fatty acids and cardiovascular disease: A systematic review. Br. J. Nutr..

[54] Fappi A., Godoy T.S., Maximino J.R., Rizzato V.R., Neves J.d.C., Chadi G., Zanoteli E. (2014). The effects of omega-3 fatty acid supplementation on dexamethasone-induced muscle atrophy. Biomed. Res. Int..

[55] Surette M.E. (2008). The science behind dietary omega-3 fatty acids. CMAJ.

[56] Shin S.K., Kim J.H., Lee J.H., Son Y.H., Lee M.W., Kim H.J., Noh S.A., Kim K.P., Kim I.G., Lee M.J. (2017). Docosahexaenoic acid-mediated protein aggregates may reduce proteasome activity and delay myotube degradation during muscle atrophy in vitro. Exp. Mol. Med..

[57] Fappi A., Neves J.d.C., Kawasaki K.A., Bacelar L., Sanches L.N., P da Silva F., Larina-Neto R., Chadi G., Zanoteli E. (2019). Omega-3 multiple effects increasing glucocorticoid-induced muscle atrophy: Autophagic, AMPK and UPS mechanisms. Physiol. Rep..

[58] Smith G.I., Atherton P., Reeds D.N., Mohammed B.S., Rankin D., Rennie M.J., Mittendorfer B. (2011). Omega-3 polyunsaturated fatty acids augment the muscle protein anabolic response to hyperinsulinaemia-hyperaminoacidaemia in healthy young and middle-aged men and women. Clin. Sci..

[59] Smith G.I., Atherton P., Reeds D.N., Mohammed B.S., Rankin D., Rennie M.J., Mittendorfer B. (2011). Dietary omega-3 fatty acid supplementation increases the rate of muscle protein synthesis in older adults: A randomized controlled trial. Am. J. Clin. Nutr..

[60] Smith G.I., Julliand S., Reeds D.N., Sinacore D.R., Klein S., Mittendorfer B. (2015). Fish oil-derived n-3 PUFA therapy increases muscle mass and function in healthy older adults. Am. J. Clin. Nutr..

[61] Gao J., Lin H., Wang X.J., Song Z.G., Jiao H.C. (2010). Vitamin E supplementation alleviates the oxidative stress induced by dexamethasone treatment and improves meat quality in broiler chickens. Poult. Sci..

[62] Balboa E., Saavedra F., Cea L.A., Ramírez V., Escamilla R., Vargas A.A., Regueira T., Sáez J.C. (2020). Vitamin E Blocks Connexin Hemichannels and Prevents Deleterious Effects of Glucocorticoid Treatment on Skeletal Muscles. Int. J. Mol. Sci..

[63] Uchitomi R., Oyabu M., Kamei Y. (2020). Vitamin D and Sarcopenia: Potential of Vitamin D Supplementation in Sarcopenia Prevention and Treatment. Nutrients.

[64] Lappe J.M., Binkley N. (2015). Vitamin D and Sarcopenia/Falls. J. Clin. Densitom..

[65] Dzik K.P., Skrobot W., Kaczor K.B., Flis D.J., Karnia M.J., Libionka W., Antosiewicz J., Kloc W., Kaczor J.J. (2019). Vitamin D Deficiency Is Associated with Muscle Atrophy and Reduced Mitochondrial Function in Patients with Chronic Low Back Pain. Oxidative Med. Cell. Longev..

[66] Hirose Y., Onishi T., Miura S., Hatazawa Y., Kamei Y. (2018). Vitamin D Attenuates FOXO1-Target Atrophy Gene Expression in C_2_C_12_ Muscle Cells. J. Nutr. Sci. Vitaminol..

[67] Beaudart C., Buckinx F., Rabenda V., Gillain S., Cavalier E., Slomian J., Petermans J., Reginster J.-Y., Bruyère O. (2014). The Effects of Vitamin D on Skeletal Muscle Strength, Muscle Mass, and Muscle Power: A Systematic Review and Meta-Analysis of Randomized Controlled Trials. J. Clin. Endocrinol. Metab..

[68] Karnia M.J., Korewo D., Myślińska D., Ciepielewski Z.M., Puchalska M., Konieczna-Wolska K., Kowalski K., Kaczor J.J. (2021). The Positive Impact of Vitamin D on Glucocorticoid-Dependent Skeletal Muscle Atrophy. Nutrients.

[69] Ricciardi C.J., Bae J., Esposito D., Komarnytsky S., Hu P., Chen J., Zhao L. (2015). 1,25-Dihydroxyvitamin D3/vitamin D receptor suppresses brown adipocyte differentiation and mitochondrial respiration. Eur. J. Nutr..

[70] Consiglio M., Viano M., Casarin S., Castagnoli C., Pescarmona G., Silvagno F. (2015). Mitochondrial and lipogenic effects of vitamin D on differentiating and proliferating human keratinocytes. Exp. Dermatol..

[71] Srikuea R., Hirunsai M., Charoenphandhu N. (2020). Regulation of vitamin D system in skeletal muscle and resident myogenic stem cell during development, maturation, and ageing. Sci. Rep..

[72] Aubry E.M., Odermatt A. (2009). Retinoic acid reduces glucocorticoid sensitivity in C_2_C_12_ myotubes by decreasing 11beta-hydroxysteroid dehydrogenase type 1 and glucocorticoid receptor activities. Endocrinology.

[73] Takisawa S., Funakoshi T., Yatsu T., Nagata K., Aigaki T., Machida S., Ishigami A. (2019). Vitamin C deficiency causes muscle atrophy and a deterioration in physical performance. Sci. Rep..

[74] Kupisz-Urbanska M., Pludowski P., Marcinowska-Suchowierska E. (2021). Vitamin D Deficiency in Older Patients—Problems of Sarcopenia, Drug Interactions, Management in Deficiency. Nutrients.

[75] Rusińska A., Pludowski P., Walczak M., Borszewska-Kornacka M., Bossowski A., Chlebna-Sokol D., Czech-Kowalska J., Dobrzańska A., Franek E., Helwich E. (2018). Vitamin D Supplementation Guidelines for General Population and Groups at Risk of Vitamin D Deficiency in Poland—Recommendations of the Polish Society of Pediatric Endocrinology and Diabetes and the Expert Panel With Participation of National Specialist Consultants and Representatives of Scientific Societies—2018 Update. Front. Endocrinol..

[76] Okazaki R., Ozono K., Fukumoto S., Inoue D., Yamauchi M., Minagawa M., Michigami T., Takeuchi Y., Matsumoto T., Sugimoto T. (2017). Assessment criteria for vitamin D deficiency/insufficiency in Japan: Proposal by an expert panel supported by the Research Program of Intractable Diseases, Ministry of Health, Labour and Welfare, Japan, the Japanese Society for Bone and Mineral Research and the Japan Endocrine Society [Opinion]. J. Bone Min. Metab..

[77] El Hajj C., Fares S., Chardigny J.M., Boirie Y., Walrand S. (2018). Vitamin D supplementation and muscle strength in pre-sarcopenic elderly Lebanese people: A randomized controlled trial. Arch. Osteoporos..

[78] Cangussu L.M., Nahas-Neto J., Orsatti C.L., Bueloni-Dias F.N., Nahas E.A. (2015). Effect of vitamin D supplementation alone on muscle function in postmenopausal women: A randomized, double-blind, placebo-controlled clinical trial. Osteoporos. Int..

[79] Suebthawinkul C., Panyakhamlerd K., Yotnuengnit P., Suwan A., Chaiyasit N., Taechakraichana N. (2018). The effect of vitamin D2 supplementation on muscle strength in early postmenopausal women: A randomized, double-blind, placebo-controlled trial. Climacteric.

[80] Meng S.-J., Yu L.-J. (2010). Oxidative Stress, Molecular Inflammation and Sarcopenia. Int. J. Mol. Sci..

[81] Santos S., Silva E., Caris A., Lira F., Tufik S., Thomatieli-Santos R. (2016). Vitamin E supplementation inhibits muscle damage and inflammation after moderate exercise in hypoxia. J. Hum. Nutr. Diet..

[82] Labonte M., Dionne I.J., Bouchard D.R., Senechal M., Tessier D., Khalil A., Labonte M., Bobeuf F., Tessier D., Khalil A. (2008). Effects of antioxidant supplements combined with resistance exercise on gains in fat-free mass in healthy elderly subjects: A pilot study. J. Am. Geriatr. Soc..

[83] Schroder H., Navarro E., Mora J., Galiano D., Tramullas A. (2001). Effects of alpha-tocopherol, beta-carotene and ascorbic acid on oxidative, hormonal and enzymatic exercise stress markers in habitual training activity of professional basketball players. Eur. J. Nutr..

[84] Gupta U., Gupta S. (2014). Sources and Deficiency Diseases of Mineral Nutrients in Human Health and Nutrition: A Review. Pedosphere.

[85] Son Y.H., Jang E., Kim Y., Lee J.-H. (2017). Sulforaphane prevents dexamethasone-induced muscle atrophy via regulation of the Akt/Foxo1 axis in C2C12 myotubes. Biomed. Pharmacother. Biomed. Pharmacother..

[86] Veronese N., Berton L., Carraro S., Bolzetta F., De Rui M., Perissinotto E., Toffanello E., Bano G., Pizzato S., Miotto F. (2014). Effect of oral magnesium supplementation on physical performance in healthy elderly women involved in a weekly exercise program: A randomized controlled trial. Am. J. Clin. Nutr..

[87] Johansson P., Dahlstrom O., Dahlstrom U., Alehagen U. (2015). Improved Health-Related Quality of Life, and More Days out of Hospital with Supplementation with Selenium and Coenzyme Q10 Combined. Results from a Double Blind, Placebo-Controlled Prospective Study. J. Nutr. Health Aging.

[88] Moslehi N., Vafa M.R., Sarrafzadeh J., Rahimi-Foroushani A. (2013). Does Magnesium Supplementation Improve Body Composition and Muscle Strength in Middle-Aged Overweight Women? A Double-Blind, Placebo-Controlled, Randomized Clinical Trial. Biol. Trace Elem. Res..

[89] Petermann-Rocha F., Chen M., Gray S., Ho F., Pell J., Celis-Morales C. (2020). Factors associated with sarcopenia: A cross-sectional analysis using UK Biobank. Maturitas.

[90] Verlaan S., Aspray T., Bauer J., Cederholm T., Hemsworth J., Hill T., McPhee J., Piasecki M., Seal C., Sieber C. (2015). Nutritional status, body composition, and quality of life in community-dwelling sarcopenic and non-sarcopenic older adults: A case-control study. Clin. Nutr..

[91] Ter Borg S., de Groot L.C., Mijnarends D.M., de Vries J.H., Verlaan S., Meijboom S., Luiking Y.C., Schols J.M. (2016). Differences in Nutrient Intake and Biochemical Nutrient Status Between Sarcopenic and Nonsarcopenic Older Adults-Results From the Maastricht Sarcopenia Study. J. Am. Med. Dir. Assoc..

[92] Chen Y.-Y., Kao T.-W., Chou C.-W., Wu C.-J., Yang H.-F., Lai C.-H., Wu L.-W., Chen W.-L. (2018). Exploring the Link between Serum Phosphate Levels and Low Muscle Strength, Dynapenia, and Sarcopenia. Sci. Rep..

